# A very rare complication of brucellosis

**DOI:** 10.1590/S1677-5538.IBJU.2015.0641

**Published:** 2016

**Authors:** Bulent Petik

**Affiliations:** 1 Department of Radiology, Adiyaman University Medical Faculty, Adiyaman, Turkey

## Testicular abscess: a very rare complication of brucellosis

**Figure 1:**
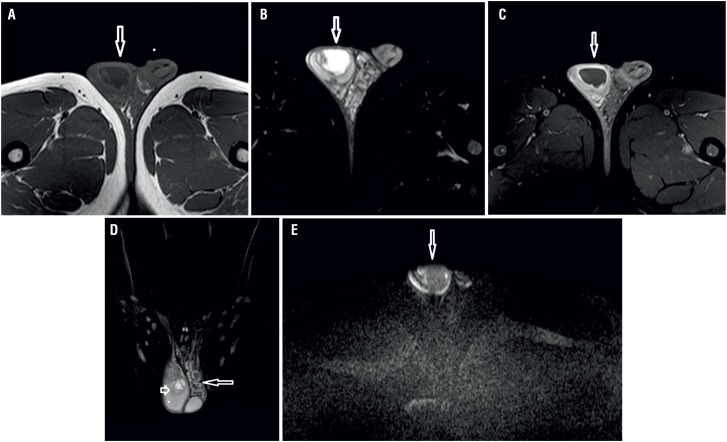
A 26-year-old male patient. On T1 weighted image (a) a hypointense cystic lesion with a thick wall is seen within the right testicle. The lesion appears hyperintense (arrow) on T2-weighted image (b). Lesion's wall shows marked enhancement after intravenous gadolinium injection (arrow) (c). Coronal T2 weighted image (d) shows the same lesion and contralateral varicocele. on diffusion weighted image obtained with a b value of 1000s/mm2 (e), the lesion shows restricted diffusion and appears hyperintense (arrow).

Our first case was a 26-year-old man presenting with right scrotal swelling, pain and redness. Sonographic evaluation revealed a cystic lesion of approximately four centimeters with a thick wall, septa, and solid components. On MR imaging, the lesion appeared hypointense on T1 weighted images with a hypointense peripheral halo surrounding its wall (Figure-1a). On T2 weighted images, the lesion was hyperintense and had a lobulated smooth contour (Figure-1b). On gadolinium-enhanced T1 weighted images, peripheral enhancement was evident (Figure-1c). Testicular abscess was associated with contralateral varicocele (Figure-1d). On diffusion weighted MR imaging, the lesion showed mild restricted diffusion on images with a b value of 500s/mm^2^ and markedly restricted diffusion on images with b values of 1000s/mm^2^, 1500s/mm^2^ and 2000s/mm^2^. The ADC value of the lesion was 1.44mm^2^/s (Figure-1e). Serologic Brucella Rose Bengal test was positive and Wright test was positive at 1/1280 titer. The patient was treated with antibiotic therapy and abscess drainage.

**Figure 2:**
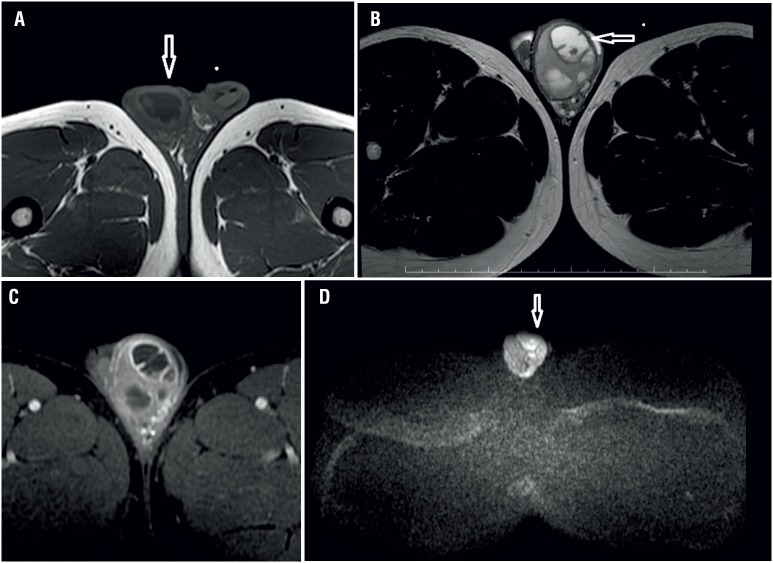
A 23-year-old male patient. On T1 weighted image a cystic lesion with heterogeneous signal intensity and thick wall is seen within the left testicle; (a). The lesion appears heterogeneous hyperintense on T2 weighted image; (b) and its wall shows enhancement; (c) after intravenous gadolinium injection. On diffusion weighted image obtained with a b value of 1000s/mm^2^; (d), the lesion shows restricted diffusion and appears hyperintense.

Our second case was a 23-year-old man with left scrotal swelling, pain and redness who was referred for scrotal MRI following a scrotal sonography that revealed a hypo echoic mass lesion with cystic areas and calcifications. On T1-weighted images, there was a septated hypointense mass lesion of approximately 3 centimeters within the left testis (Figure-2a). The lesion was hyperintense on T2-weighted images (Figure-2b) and showed peripheral enhancement on T1-weighted gadolinium enhanced images (Figure-2c). On diffusion weighted MR imaging, the lesion showed mild restricted diffusion on images with a b value of 500s/mm^2^ and markedly restricted diffusion on images with b values of 1000s/mm^2^, 1500s/mm^2^ and 2000s/mm^2^. The ADC value of the lesion was 1.165mm^2^/s (Figure-2d). Wright test was positive at 1/160 titer. The patient was treated with antibiotic therapy and abscess drainage.

**Figure 3:**
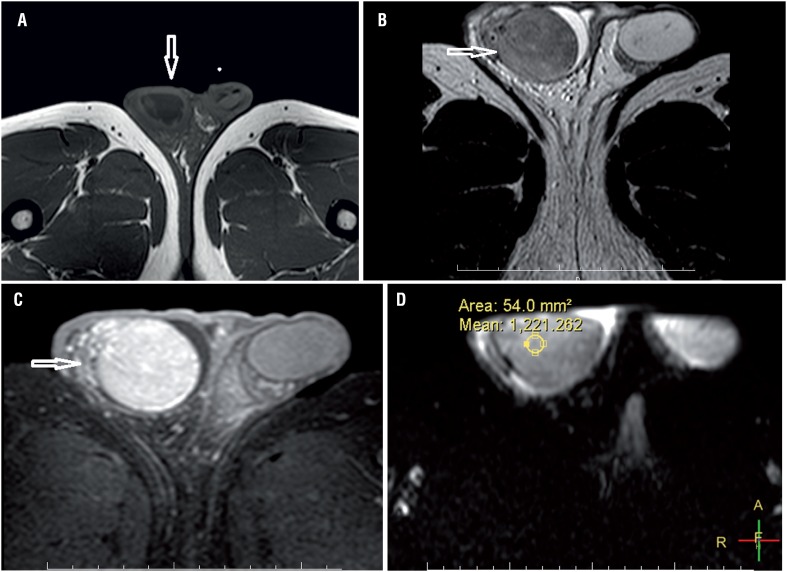
A 24-year-old male patient. On T1 weighted image the right testis was slightly hyperintense relative to left testis and enlarged; (a). The right testis appears hypointense compared to left testis on t2 weighted image (arrow); (b). After intravenous gadolinium injection and it shows strong enhancement; (c). The lesion has an ADC value of 1.221mm2/s (d).

Our third case was a 24-year-old man with painless right scrotal swelling. Sonography revealed an enlarged right testis with a diffusely hypoechoic nodular appearance. On T2-weighted MR images (Figure-3a) right testis was hypointense relative to the left testis. Right testis appeared slightly hyperintense on T1-weighted images (Figure-3b) when compared with the left side and showed strong enhancement after intravenous injection of gadolinium (Figure-3c). The lesion showed mild restricted diffusion on images with a b value of 500s/mm^2^, 1000s/mm^2^, 1500s/mm^2^ 1500s/mm^2^ and 2000s/mm^2^. The ADC value of the lesion was 1.221mm^2^/s (Figure-3d). Wright test was positive at 1/320 titer. The patient was treated with antibiotic therapy and abscess drainage.

Regarding the scrotal and testicular masses, it is of crucial importance to correctly characterize the lesion to avoid unnecessary orchiectomies (1, 2). Specifically for patients with abscesses due to testicular brucellosis, the main differential diagnosis is necrotic tumors that may necessitate orchiectomy. On the other hand, failure to correctly diagnose a testicular brucellosis abscess may lead to delayed treatment and partial or total destruction of the testis.

Testicular brucellosis abscesses occur in patients with epididymo-orchitis when necrosis develops in the region of granulomatous infection induced by the persistence of the bacteria in macrophages (3). A testicular brucellosis abscess is an extremely rare entity with nine published cases in the literature so far. Of these nine cases, three patients underwent conservative treatment with antibiotics and drainage (3). The remaining six patients underwent orchiectomy with combined antibiotic treatment (3). Three cases we present in this study were treated with antibiotic therapy and drainage, as well.

In conclusion, brucellosis testicular abscesses are lesions that need to be diagnosed precisely to plan the most appropriate therapy. Sonography is very useful to detect these lesions. To correctly characterize these rare lesions, contrast enhanced scrotal MRI combined with diffusion weighted sequences is needed.

